# Identification of Multiple Novel Viruses in Fecal Samples of Black-Necked Cranes Using Viral Metagenomic Methods

**DOI:** 10.3390/v15102068

**Published:** 2023-10-09

**Authors:** Qifan Zhao, Ran Zhao, Yijie Sun, Li Ji, Yuan Xi, Xiaochun Wang, Quan Shen, Likai Ji, Yan Wang, Zhenqiang You, Shixing Yang, Wen Zhang

**Affiliations:** 1 Department of Laboratory Medicine, School of Medicine, Jiangsu University, Zhenjiang 212013, China; q351461127@gmail.com (Q.Z.); sunyijie111@163.com (Y.S.); 13952877636@163.com (L.J.); 2212113096@stmail.ujs.edu.cn (Y.X.); chun_xiao@163.com (X.W.); shenquanfly@yahoo.com (Q.S.); jilikai01@ujs.edu.cn (L.J.); wangyan_jtu@126.com (Y.W.); 2 Department of Prevention and Control, Xiamen Animal Disease Prevention and Control Center, Xiamen 361009, China; xzzhaoran@163.com; 3 School of Public Health, Hangzhou Medical College, Hangzhou 310013, China; youzq1979@163.com

**Keywords:** viral metagenomics, black-necked crane, genomic structure, phylogenetic analysis

## Abstract

The black-necked crane is the only species of crane that lives in the high-altitude region of the Tibet Plateau. At present, there is little research on viral diseases of the black-necked crane (*Grus nigricollis*). In this study, a viral metagenomic approach was employed to investigate the fecal virome of black-necked cranes in Saga County, Shigatse City, Tibet, China. The identified virus families carried by black-necked cranes mainly include *Genomoviridae*, *Parvoviridae*, and *Picornaviridae*. The percentages of sequence reads belonging to these three virus families were 1.6%, 3.1%, and 93.7%, respectively. Among them, one genome was characterized as a novel species in the genus *Grusopivirus* of the family *Picornaviridae*, four new parvovirus genomes were obtained and classified into four different novel species within the genus *Chaphamaparvovirus* of the subfamily *Hamaparvovirinae*, and four novel genomovirus genomes were also acquired and identified as members of three different species, including *Gemykroznavirus haeme1*, *Gemycircularvirus ptero6*, and *Gemycircularvirus ptero10*. All of these viruses were firstly detected in fecal samples of black-necked cranes. This study provides valuable information for understanding the viral community composition in the digestive tract of black-necked cranes in Tibet, which can be used for monitoring, preventing, and treating potential viral diseases in black-necked cranes.

## 1. Introduction

The black-necked crane is a vulnerable species and the only species of crane that inhabits the Tibet Plateau. It is classified as a vulnerable species (VU) by the International Union for Conservation of Nature (IUCN), with a global population of about 17,000 individuals [1]. The Tibet Plateau is one of the main habitats for black-necked cranes, containing one of the largest populations of this species in the world. Every year, approximately 11,000 black-necked cranes overwinter and breed here. They live in the freshwater fields of the Tibet Plateau and feed on plant roots, insects, fish, frogs, and residual crop seeds found on farmland. However, human activities, including agriculture, mining, and tourism, are increasingly threatening the black-necked crane population [2,3,4]. Additionally, viruses that infect birds and other crane species are potentially endangering their lives. In recent years, emerging and re-emerging viruses, such as avian influenza virus, Marek’s disease virus, West Nile virus, and crane hepatitis herpesviruses, have been found to infect cranes, posing health risks [5,6,7,8]. Some of these viruses also potentially cross species barriers and infect humans. Nevertheless, there have been limited virological studies conducted on black-necked cranes.

Viral metagenomics is a powerful tool for exploring both new and known viruses, and it has been widely utilized to understand viral compositions in diverse samples [9,10]. However, the viral composition in fecal samples from black-necked cranes remains poorly understood. Therefore, the objective of this study is to investigate the viral composition of fecal samples collected from black-necked cranes in their natural habitat in Sa’gya County, Tibet Province, China, using a viral metagenomic approach. The findings of this study will provide valuable genome information for the prevention and treatment of potential viral diseases in this vulnerable species.

## 2. Materials and Methods

### 2.1. Sample Collection and Preparation

In December 2020, 10 fecal samples were collected from healthy black-necked cranes in Sakya County, Shigatse City, Tibet Province, China. For sampling, all black-necked cranes were captured using cannon nets. Fecal samples were collected by using disposable, absorbent cotton swabs and were shipped to our laboratory on dry ice. About two grams of each fecal sample was resuspended in 2 mL of phosphate-buffered saline (PBS) and vigorously vortexed for 10 min and then centrifuged at 12,000 rpm for 10 min. Finally, each fecal supernatant was collected in a new 1.5 mL centrifuge tube and stored at −80 °C for further use.

### 2.2. Viral Nucleic Acid Extraction

A total of 500 μL of fecal suspension (50 μL of fecal supernatant from each fecal sample) was pooled together and filtered through a 0.45 µm filter (Merck Millipore, Billerica, MA, USA) to remove bacterial and eukaryotic cell-sized particles. The filtrates were then treated with a mixture of nuclease enzymes to digest the unprotected nucleic acids at 37 °C for 90 min. Viral RNA and DNA were extracted by using the QIAamp MinElute Virus Spin Kit (Qiagen, Hilden, NRW, Germany) according to the manufacturer’s improved protocol. The concentrations of DNA and RNA were calculated using the Qubit 4 (Invitrogen, Carlsbad, CA, USA) nucleic acid concentration sequencer.

### 2.3. Library Construction and Bioinformatics Analysis

The cDNA of viral RNA was synthesized by using reverse transcription using six-base random primers. The complementary chain of cDNA was generated using Klenow Fragment DNA polymerase (M0210L, New England Biolabs, Rowley, MA, USA). Next, libraries were constructed using the Nextera XT DNA Sample Preparation Kit (Illumina, San Diego, CA, USA) and were sequenced using the NovaSeq Illumina platform with 250 base-paired ends with dual barcoding for each pool.

For bioinformatics analysis, the paired-end reads of 250 bp generated by NovaSeq were debarcoded using the vendor software from Illumina. We used an in-house analysis pipeline running on a 32-node Linux cluster to process the data. Low-sequencing quality tails were trimmed using a Phred quality score threshold of 10. Adaptors were trimmed using the default parameters of VecScreen, which is an NCBI BLASTn program with specialized parameters designed for adaptor removal. Bacterial reads were subtracted by mapping them to bacterial nucleotide sequences from the BLAST NT database using Bowtie2 v2.2.4. The cleaned reads were then de novo assembled by SOAPdenovo2 version r240 using a Kmer size of 63 with default settings [11]. The assembled contigs, along with singlets, were aligned to an in-house viral proteome database using BLASTx (v.2.2.7) with an E-value cutoff of <10^−5^. The candidate viral hits were compared to an in-house nonvirus nonredundant (NVNR) protein database to remove false-positive viral hits. The NVNR database was compiled using nonviral protein sequences extracted from the NCBI nr fasta file and was based on a taxonomy annotation excluding the virus kingdom.

### 2.4. Phylogenetic Analysis

The analysis of evolutionary relationships was carried out using amino acid sequences predicted from the genomic data, with reference to the closest viral relatives determined by the best BLASTx hit and representative members of related viral species or genera. Sequence alignment was conducted with Clustal W in MEGA version X using the default settings [12]. Phylogenetic trees were constructed using MrBayes v3.2.7, with the parameters “lset nst  =  6 rates  =  invgamma”. This setting applied the GTR substitution model with gamma-distributed rate variation across sites and used a proportion of invariable sites (“GTR  +  I + Г”). Additionally, “prset aamodelpr  =  mixed” was employed to enable the program to use the ten built-in amino acid models. The maximum number of generations was set to be ten million, and sampling occurred at every 50 generations, with the first 25% of Markov chain Monte Carlo (mcmc) samples being discarded during burn-in. Convergence was confirmed when the standard deviation of split frequencies was below 0.01. Bootstrap values were assigned to each node.

### 2.5. Sequence Alignment and ORF Prediction

The pairwise comparison of viral amino acid sequences was conducted using SDTv1.2 software with default settings. Putative open reading frames (ORFs) in the genome were predicted using Geneious 11.1.2 software and the NCBI ORF finder. For genomoviruses, putative exons and introns were predicted using the Netgenes2 online website (https://services.healthtech.dtu.dk/services/NetGene2-2.42/) (accessed on 20 August 2023).

### 2.6. Nucleotide Sequence Accession Number

The viral genome sequences were deposited in the GenBank with the accession numbers from OR532946 to OR532954. The raw sequence reads from the metagenomic library were deposited in the Shirt Read Archive of the GenBank database under accession number SRR25662272.

## 3. Results

### 3.1. Viral Metagenomic Overview

**Figure 1:**
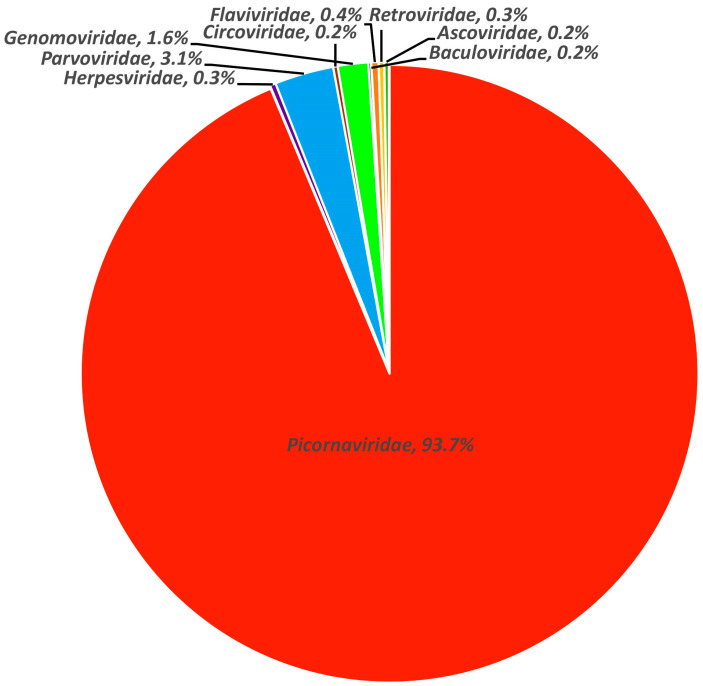
Pie chart of the composition of fecal virome detected in black-necked cranes, shown as percentages. The percentage of sequence reads in different viral families refers to all viruses in the same viral family obtained from the library and not just new viruses that were identified.

The library generated a total of 4,837,834 raw sequence reads on the Illumina NovaSeq platform. After conducting a bioinformatics analysis, it was found that 139,584 sequence reads had the best matches with viral proteins. This accounted for 2.9% of the total number of raw data reads. Further analysis was conducted to determine the percentage of viral reads belonging to different virus families. Among them, sequence reads from the *Picornaviridae* family accounted for the largest proportion, representing 93.7% of the total analyzed virus reads. This was followed by the *Parvoviridae* family at 3.1% and the *Genomoviridae* family at 1.6%. The remaining virus families, including *Herpesviridae*, *Circoviridae*, *Baculoviridae*, *Flaviviridae*, *Ascoviridae*, and *Reoviridae*, accounted for a small proportion (Figure 1).

### 3.2. A Novel Picornavirus Belonging to the Genus Grusopivirus of the Family Picornaviridae

**Figure 2:**
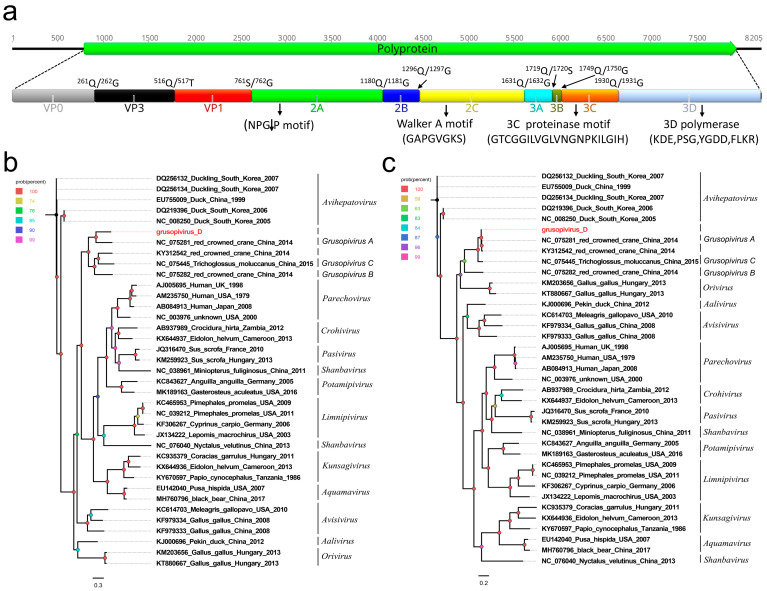
The genomic organization, conserved motifs, and phylogenetic analysis of the grusopivirus identified in black-necked cranes. (**a**) The genomic organization of one grusopivirus strain. The ORF- and viral-encoding proteins of grusopivirus are marked with different colors. The conserved motifs are also shown. (**b**,**c**) Phylogenetic analysis based on the P1 region and 3CD of grusopivirus, which was identified in this study, and other reference strains belonging to the subfamily *Paavivirinae* of the family *Picornaviridae*. Grusopivirus D, which was identified in this study, is highlighted in red.

In this study, 79,484 sequence reads belonging to the family *Picornaviridae* were found in the library, where 74,237 sequence reads were matched with the same picornavirus using the ‘Map to Reference’ program in Geneious 11.1.2. One nearly complete genome of picornavirus was obtained using the assemble sequences program in Geneious 11.1.2. A blastn comparison of the genome showed that the new picornavirus was closely related (90.06%) to other members of the genus *Grusopivirus*, and it is tentatively named Grusopivirus D. The genome of Grusopivirus D is 8205 nt in length, which includes a 789 nt 5′ UTR, a 7155 nt polyprotein ORF, and a 261 nt 3′ UTR. The GC content of Grusopivirus D is 41.8%. Similar to the members of avihepatoviruses and parechoviruses, the polyprotein of Grusopivirus D can be divided into VP0, VP3, VP1, 2A–2C, and 3A–3D via a comparison with the polyprotein of the Avihepatovirus A strain (NC_008250) and Parechovirus A strain (AB084913) (Figure 2a). The P1 polypeptide is 761 aa in length and cleaved at VP0/VP3 (Q^261^/G^262^) and VP3/VP1 (Q^516^/T^517^). It shared the highest amino acid identity of 40.81% with that of the Grusopivirus A1 strain (NC_075281). The P2 polypeptide of 870 aa contains three nonstructural proteins including 2A (cleavage site: Q^1180^/G^1181^), 2B (cleavage site: Q^1296^/G^1297^), and 2C. BLASTp showed that the P2 region shared a 96.21% amino acid sequence identity with that of the Grusopivirus A1 strain (NC_075281). A conserved NPGP motif function mediating the cotranslational termination–reinitiation of RNA translation was present in the 2A protein, while the conserved NTPase motif “GAPGVGKS” was also found in its 2C protein (Figure 2a). The P3 polypeptide is 753 aa in length and cleaved into four nonstructural proteins including 3A, 3B, 3C^pro^ (protease), and 3D^pol^ (RNA-dependent RNA polymerase) at sites 3A/3B (Q^1719^/S^1720^), 3B/3C (Q^1749^/G^1750^), and 3C/3D (Q^1930^/G^1931^). The P3 of Grusopivirus D shared the highest amino acid sequence identity of 98.41% with that of the Grusopivirus A1 strain (NC_075281). Multiple conserved proteinase and polymerase motifs, including GXCGX_10–15_GXH, KDE, PSG, YGDD, and FLKR, were separately found in the 3C and 3D proteins (Figure 2a).

Two phylogenetic trees were constructed based on the P1 region and 3CD of Grusopivirus D and on 36 other representative strains belonging to the subfamily *Paavivirinae* of the family *Picornaviridae* (Figure 2b). The results show that Grusopivirus D clusters with the strains NC_075281, KY312542, NC_075445, and NC_075282 and forms a separate clade. Among them, Grusopivirus D had the closest genetic distance with the strain NC_075281, which was isolated from the fecal sample of red-crowned cranes in 2014 [13]. The P1 region of Grusopivirus D shares a 40.81% amino acid sequence identity with that of the strain NC_075281, and its polyprotein is 178 aa longer than that of the strain NC_075281. According to the International Committee on Taxonomy of Viruses (https://ictv.global/report/chapter/picornaviridae/picornaviridae) (accessed on 21 August 2023), members of a picornavirus species should share a significant degree of amino acid identity in the P1, 2C, 3C, and 3D proteins. Our result indicates that Grusopivirus D should be defined as a novel species in the genus *Grusopivirus*.

### 3.3. Four New Parvoviruses Belonging to the Genus Chaphamaparvovirus of the Family Parvoviridae

**Figure 3:**
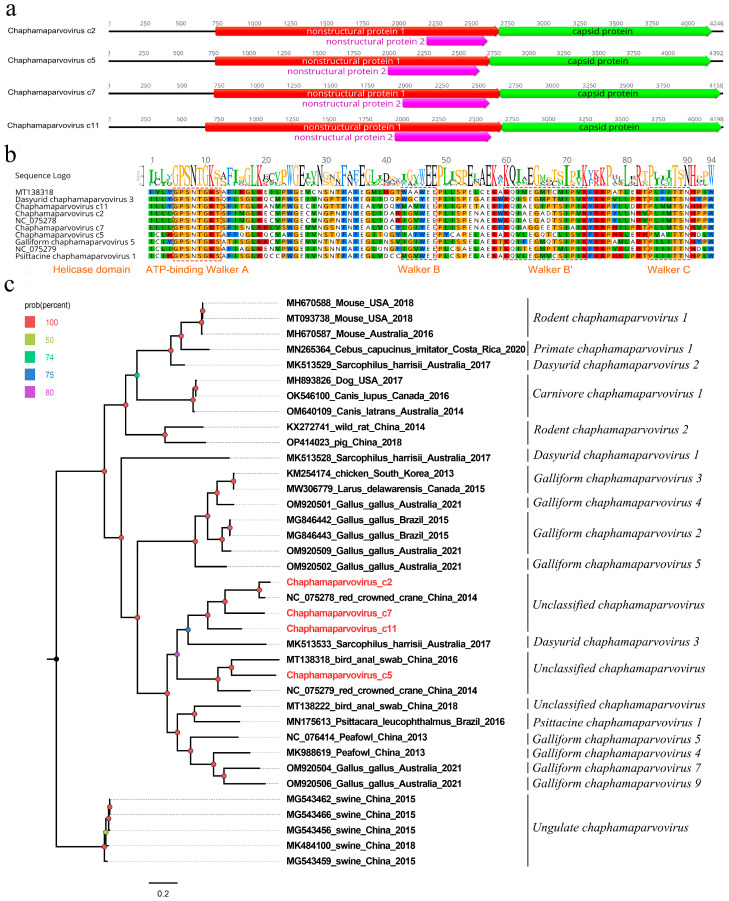
The genomic organization, conserved helicase domain motifs, and phylogenetic analysis of four chaphamaparvoviruses identified in black-necked cranes. (**a**) The genomic organization of four chaphamaparvoviruses identified in black-necked cranes. Viral-encoding proteins of four chaphamaparvoviruses are marked with different colors, in which red is used for nonstructural protein 1, pink for nonstructural protein 2, and green for capsid protein. (**b**) The conserved NTPase/helicase motif (Walker A, B, B’, and C) of these four parvoviruses and other reference strains are shown. The conserved motifs are marked with an orange frame. (**c**) The phylogenetic analysis was based on NS1 proteins of the mentioned parvoviruses, as well as on their closest viral relatives, which were determined using the best BLASTp hits and other representative strains belonging to the genus *Chaphamaparvovirus* of the subfamily *Hamaparvovirinae*. The four chaphamaparvoviruses identified in this study are highlighted in red.

Here, 2618 sequence reads assigned to the *Parvoviridae* family were detected in the library, where 910, 730, 360, and 185 sequence reads were matched with the four parvoviruses. Four nearly complete genomes of parvoviruses were acquired using the assemble sequences program in Geneious 11.1.2. A blastn comparison of the genomes showed that the four parvoviruses were closely related (84.99%, 66.46%, 66.95%, and 67.96%) to other members of the genus *Chaphamaparvovirus*, and they are temporarily named Chaphamaparvovirus c2, c5, c7, and c11. The genomes of Chaphamaparvovirus c2, c5, c7, and c11 are 4246 nt, 4392 nt, 4158 nt, and 4198 nt in length and have three ORFs encoding two nonstructural proteins (NS1 and NS2) and one structural protein (VP) (Figure 3a). The length of the NS1 protein is 654 aa for Chaphamaparvovirus c2, 657 aa for c5, 648 aa for c7, and 678 aa for c11, while the NS2 length of these four parvoviruses is 139 aa, 195 aa, 218 aa, and 219 aa, respectively. The conserved NTPase/helicase motifs (Walker A, B, B’, and C) were found in all four NS1 proteins; however, there are differences in some amino acid sites in this conserved domain compared with those of the reference chaphamaparvoviruses (NC_075279, NC_075278, MT138318, NC_076414, NC_076370, and NC_076425) [14] (Figure 3b). The VP proteins of these four viruses are different in length. Among them, the longest VP is in Chaphamaparvovirus c2 (526 aa), followed by Chaphamaparvovirus c11 (499 aa), Chaphamaparvovirus c7 (496 aa), and Chaphamaparvovirus c5 (490 aa). The conserved phospholipase A_2_ (PLA2) motif that is often present in members of the subfamily *Parvovirinae* was absent in the VP proteins of these parvoviruses [15].

A phylogenetic tree was constructed using the NS1 proteins of the mentioned parvoviruses, as well as using their closest viral relatives based on the best BLASTp hits and other representative strains belonging to the genus *Chaphamaparvovirus* of the subfamily *Hamaparvovirinae*. The result shows that Chaphamaparvovirus c2, Chaphamaparvovirus c7, and Chaphamaparvovirus c11 were clustered with the strain NC_075278, which was detected in the fecal sample of red-crowned cranes, forming a separate clade [13]. Additionally, Chaphamaparvovirus c5 was clustered with the strain MT138318, which was isolated from *Grus grus*, forming another separate clade [9] (Figure 3c). To determine the species or genus of these parvoviruses, a pairwise comparison of NS1 was conducted. The result shows that Chaphamaparvovirus c2 shared a >87% amino acid sequence identity with the strain NC_075278, which is an unclassified chaphamaparvovirus, while Chaphamaparvovirus c5, Chaphamaparvovirus c7, and Chaphamaparvovirus c11 shared a <60% but >35% amino acid sequence identity with other members of the genus *Chaphamaparvovirus* or with each other (Figure S1). Based on the novel demarcation criteria proposed by Judit J. Pénzes and coworkers, viruses can be considered members of the same species if the NS1 proteins share more than an 85% amino acid sequence identity [16]. Therefore, the four parvoviruses identified here belong to four different novel species within the genus *Chaphamaparvovirus* of the subfamily *Hamaparvovirinae*.

### 3.4. Four Novel Genomoviruses Belonging to the Family Genomoviidae

**Figure 4:**
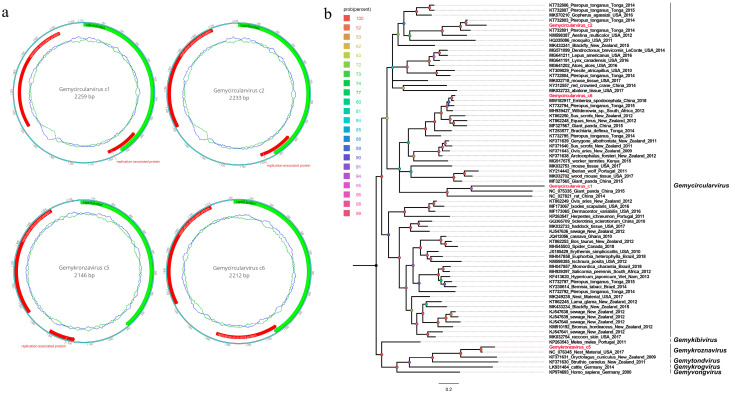
The genomic organization and phylogenetic analysis of genomoviruses identified in black-necked cranes. (**a**) The genomic organization of four genomoviruses identified in black-necked cranes. Viral-encoding proteins of four genomoviruses are marked with different colors, in which red is used for replication-associated protein and green for capsid protein. (**b**) Phylogenetic analysis based on the Rep proteins of four genomoviruses identified in this study and on reference strains of other genomoviruses. The four genomoviruses identified in this study are highlighted in red.

In this study, 1341 sequence reads belonging to the family *Genomoviridae* were found in the library, where all sequence reads were matched with the four genomoviruses. Four complete genomes of genomoviruses were obtained using the assemble sequences program in Geneious 11.1.2. A blastn comparison of the genomes showed that three of the genomoviruses were closely related (92.11%, 93.76%, and 87.85%) to other members of the genus *Gemycircularvirus*, and one genomovirus was closely related (96%) to other members of the genus *Gemykronznavirus*; thus, they are named Gemycircularvirus c1, Gemycircularvirus c2, Gemycircularvirus c6, and Gemykronzavirus c5, respectively. Their genomes are 2259 nt, 2233 nt, 2146 nt, and 2212 nt in length and have two bidirectional ORFs encoding a capsid protein (CP) and a replicase-associated protein (Rep). An intron lies within the Rep gene, which is similar to those in some genomoviruses (Figure 4a). The length of the CP protein is 309 aa for both Gemycircularvirus c1 and c2, 314 aa for Gemycircularvirus c6, and 285 aa for Gemykronzavirus c5. The Rep protein of these four genomoviruses is 269 aa for Gemycircularvirus c1, 261 aa for Gemycircularvirus c2, 239 aa for Gemykronzavirus c5, and 304 aa for Gemycircularvirus c6. The conserved rolling-circle replication (PCR) motifs I, II, and III were present in the Rep proteins of Gemycircularvirus c2 (FTYSQ, HFHVFTD, and YAIKD), Gemykronzavirus c5 (LTYSQ, HYHVVAQ, and YCLKD), and Gemycircularvirus c6 (LTYSQ, HLHVFCD, and YATD), while in the Rep protein of Gemycircularvirus c1, only PCR motif I (FTYSQ) was found and not motif II and III.

A phylogenetic tree was constructed based on these Rep proteins and on their closest viral relatives, which were determined using the best BLASTp hits and other representative strains belonging to several different genera of the family *Genomoviridae*. The result shows that these four genomoviruses were located on four separate branches; among them, Gemycircularvirus c2 was clustered with the Pteropus-associated gemycircularvirus (GenBank no. KT732801), which was detected in the fecal sample of Pteropus tonganus in Tonga; Gemycircularvirus c6 was clustered with the Emberiza spodocephala gemycircularvirus (GenBank no. MW182917), which was isolated from an anal swab of Emberiza spodocephala in China; Gemycircularvirus c1 was clustered with the giant panda-associated gemycircularvirus (GenBank no. NC_075335), which was detected in the fecal sample of the giant panda in China; and Gemykronzavirus c5 was clustered with the Finch-associated genomovirus (GenBank no. NC_076345), which was found in the nest material of finches in the USA [17,18,19] (Figure 4b). To determine the species or genus of these genomoviruses, a pairwise comparison of genome-wide identities was conducted. The result shows that Gemykronzavirus c5 shared a >78% genome-wide identity with the representative strain NC_076345, which belongs to the species of *Gemykroznavirus haeme1*; Gemycircularvirus c1 and c2 shared a >78% genome-wide identity with the representative strain KT732803, belonging to the species of *Gemycircularvirus ptero6*; and Gemycircularvirus c6 shared a >78% genome-wide identity with the representative strain KT732794, belonging to the species of *Gemycircularvirus ptero10* (Figure S2). According to the novel demarcation criteria proposed by Arvind Varsani and coworkers, a genome-wide pairwise identity of 78% was chosen as the species demarcation threshold for genomoviruses, whereas Rep sequence phylogeny was used to define genera [20]. Based on these criteria, the four genomoviruses found here are classified into three species belonging to two different genera: *Gemycircularvirus* and *Gemykroznavirus*. Specifically, the three species are *Gemykroznavirus haeme1*, *Gemycircularvirus ptero6*, and *Gemycircularvirus ptero10*.

## 4. Discussion

The black-necked crane, as a vulnerable species, is highly susceptible to viral diseases. Previous studies have identified various viruses that can infect cranes and cause diseases. For instance, Lee and coworkers isolated a low-pathogenic H7N7 avian influenza virus from a red-crowned crane in a zoo in South Korea [5]. Ozawa and coworkers reported the widespread prevalence of crane-associated adenovirus 1 in cranes overwintering on the Izumi Plain, Japan [21]. Taniguchi and coworkers demonstrated that crane herpesvirus causes hemagglutination [22]. In our previous study, viral metagenomics revealed the presence of multiple viruses in the fecal samples of red-crowned cranes [13]. However, limited research has been conducted on viral diseases in black-necked cranes. Hence, in this study, we employed high-throughput sequencing to investigate the fecal virome of black-necked cranes. Our results are the first to detect multiple viruses in black-necked cranes and classify them as novel virus species.

Members of the *Picornaviridae* family are small, single-stranded RNA viruses with genome lengths ranging from approximately 7.2 to 9.4 kb. The *Picornaviridae* family currently consists of 158 species grouped into 68 genera [23]. Different genera of the family *Picornaviridae* infect various animals and humans, leading to a variety of diseases [24,25,26,27,28]. For instance, Enterovirus is the most common genus of the *Picornaviridae* virus and can cause diseases such as hand-foot-and-mouth disease, poliomyelitis, and myocarditis [29,30,31]. Hepatovirus includes human hepatitis A virus, which can cause acute hepatitis [32]. Recently, viruses in the genus *Senecavirus* have garnered attention from both veterinary and public health communities because they have been found to cause swine hand-foot-and-mouth disease [33]. *Grusopivirus*, as a novel genus of the *Picornaviridae* family, was first discovered in the fecal sample of a healthy red-crowned crane in 2014 by our lab [13]. In this study, a novel grusopivirus was detected for the first time in the fecal samples of black-necked cranes. This virus has a similar genomic structure and potential cleavage sites as the members of avihepatoviruses, which can cause poultry diseases. This suggests that the novel grusopivirus has the potential to be pathogenic in black-necked cranes. By determining amino acid sequence alignment, we found that P1 of Grusopivirus D only shared an amino acid sequence identity of 40.81% with that of the Grusopivirus A1 strain (NC_075281), while P2 and P3 of Grusopivirus D shared an amino acid sequence identity over 96% with that of Grusopivirus A1. Considering that the capsid protein, encoded by P1 of picoranvirus, plays an important role as a ligand during a viral infection within the body, we speculate that these differences in the P1 polypeptide are related to differences in host receptors. Currently, all discovered grusopiviruses have been isolated from members of the *Gruiforms* family. This prompted us to think that grusopiviruses can only infect cranes. However, the epidemiological and pathological characteristics of grusopiviruses are not well understood. Therefore, further experimental and epidemiological studies are needed to understand their pathogenesis and transmission mechanisms.

The parvovirus is a nonenveloped, icosahedral, single-stranded DNA virus with a genome that is approximately between 4 kb and 6 kb in length [34]. The *Parvoviridae* family is classified into three subfamilies, including *Densovirinae, Parvovirinae*, and *Hamaparvovirinae*. The subfamily *Hamaparvovirinae* was identified recently, which includes the genera *Brevihamaparvovirus*, *Chaphamaparvovirus*, *Hepanhamaparvovirus*, *Ichthamaparvovirus*, and *Penstylhamaparvovirus* [35]. The genus *Chaphamaparvovirus* is classified into 16 species, including *Carnivore chaphamaparvovirus 1–2*, *Chiropteran chaphamaparvovirus* 1, *Dasyurid chaphamaparvovirus 1–3*, *Galliform chaphamaparvovirus 1–5*, *Primate chaphamaparvovirus 1*, *Psittacine chaphamaparvovirus 1*, *Rodent chaphamaparvovirus 1–2*, and *Ungulate chaphamaparvovirus 1*. Members of the genus *Chaphamaparvovirus* can infect various animals, including dogs, wolves, chickens, pheasants, *Larus delawarensis* (a species of gull), bats, *Sarcophilus harrisii* (*Tasmanian devils*), *Pavo cristatus* (peacocks), Cebusimitator (white-headed capuchins), parrots, and rodents [36,37,38,39,40,41]. Some of these infections can cause diseases in their respective hosts. For example, a study by Michael et al. reported that chaphamaparvovirus was the cause of hepatitis outbreaks in pheasants (*Phasianus colchicus*), which were characterized by high mortality [38]. Subir Sarker also found that galliform chaphamaparvovirus was associated with spotty liver disease in chickens [42]. Additionally, dogs, especially puppies infected with carnivore chaphamaparvovirus, exhibited clinical signs, such as diarrhea, fever, and cough [43]. In this study, four novel chaphamaparvoviruses were characterized for the first time. They were classified into four different novel species within the genus *Chaphamaparvovirus* of the subfamily *Hamaparvovirinae*. Surprisingly, phylogenetic analysis and pairwise alignment indicated that these four chaphamaparvoviruses had a higher genetic relationship with representative strains that were detected from fecal samples of healthy red-crowned cranes than strains found in other animals. We speculate that chaphamaparvovirus is widely distributed among members of the *Gruiforms* family. Further epidemiological investigation is needed to determine whether these viruses can cause disease in black-necked cranes and whether interspecies transmission is possible.

Members of the family *Genomoviridae* are small, icosahedral, nonenveloped single-stranded circular DNA viruses. Their genomes are approximately 1.8–2.4 kb in length and encode a rolling-circle replication initiation protein (Rep) and a capsid protein (CP) in an ambisense orientation [44]. The family *Genomoviridae* is currently classified into nine genera: *Gemycircularvirus*, *Gemyduguivirus*, *Gemygorvirus*, *Gemykibivirus*, *Gemykolovirus*, *Gemykrogvirus*, *Gemykroznavirus*, *Gemytondvirus*, and *Gemyvongvirus* [15]. The genus *Gemycircularvirus* is classified into 126 species, while the genus *Gemykroznavirus* is divided into 7 species. The first discovered genomovirus was Sclerotinia sclerotiorum hypovirulence-associated DNA virus 1 (SsHADV-1), which infects the phytopathogenic fungus *Sclerotinia sclerotiorum* [45]. Recently, multiple genomoviruses have been detected in diverse samples taken from various organisms, including Actinopterygii, Arachnida, Aves, Embryophyte, Gastropoda, Insecta, Leotiomycetes, Mammalia, and Reptilia, as well as in samples taken from the environment [20,46,47,48,49,50]. In the present study, we detected four novel genomoviruses for the first time in fecal samples from black-necked cranes. From a genome evolution perspective, these four genomoviruses are genetically related to strains isolated from the nest material of finches in the USA or from feces of healthy Pteropus tonganus in Tonga. This indicates that the same species of genomovirus can infect different bird species, even if they live in different locations. However, because our samples were collected from healthy individuals, we cannot be certain if these novel genomoviruses will cause disease in black-necked cranes. It is also possible that these novel genomoviruses originated from foodborne insects. Therefore, conducting further epidemiological investigations, including a large-scale collection of blood samples, will be beneficial in clarifying whether these genomoviruses are true pathogens of black-necked cranes. For the replication of the genomovirus, the presence of the three conserved PCR motifs (I, II, and III) in the Rep protein is crucial. Surprisingly, only PCR motif I was found in the Rep protein of Gemycircularvirus c1, while motifs II and III were absent. However, the other three genomoviruses did exhibit all three conserved PCR motifs. It is unknown whether the absence of two PCR motifs will affect the proliferation of Gemycircularvirus c1. Further experiments on virus proliferation in infected cells will help answer this question. Additionally, phylogenetic analysis based on the Rep proteins revealed that Gemycircularvirus c1 and Gemycircularvirus c2 are located in different branches. Therefore, we believe that phylogenetic trees constructed based on the Rep proteins can be effectively utilized for clustering virus genera, but may not be suitable for determining virus species.

Although multiple new viruses were identified in fecal samples of black-necked cranes using a viral metagenomic method in this study, we were unable to determine whether these new viruses can cause disease in black-necked cranes. This limitation stems from the small number of samples collected and the exclusive focus on fecal samples. Moreover, we are uncertain about the exact sources of these viruses and their potential for cross-species transmission. Consequently, conducting a comprehensive epidemiological investigation in the region is crucial and necessitates the collection of various sample types from numerous animals on a larger scale.

## 5. Conclusions

In summary, this study presents an overview of the viral community found in the feces of black-necked cranes and significantly enhances our understanding of the viral composition of their stool samples. The prevalence of viruses related to the black-necked crane, as described in this study, offers valuable genome information for the prevention and treatment of potential viral diseases in birds in the local region.

## Data Availability

The viral genome sequences were deposited in the GenBank with the accession numbers OR532946 to OR532954. The raw sequence reads from the metagenomic library were deposited in the Shirt Read Archive of the GenBank database under accession number: SRR25662272.

## Supplementary Materials

The following supporting information can be downloaded at: https://www.mdpi.com/article/10.3390/v15102068/s1, Figure S1: Pairwise comparison of NS1 amino acid sequences of four chaphamaparvoviruses identified in this study with the representative strains of different species of the genus *Chaphamaparvovirus*. The pairwise identity (%) was marked with different colors. Figure S2: pairwise comparison of genome-wide nucleotide sequences of four genomoviruses identified in this study with the representative strains of different genera of the family *Genomoviridae*. The pairwise identity (%) was marked with different colors.
