# Supplementary figures and images for: Identification of Multiple Novel Viruses in Fecal Samples of Black-Necked Cranes Using Viral Metagenomic Methods
Source: Viruses. 2023 Oct 9;15(10):2068. doi: 10.3390/v15102068 (PMC10612090; doi:10.3390/v15102068)

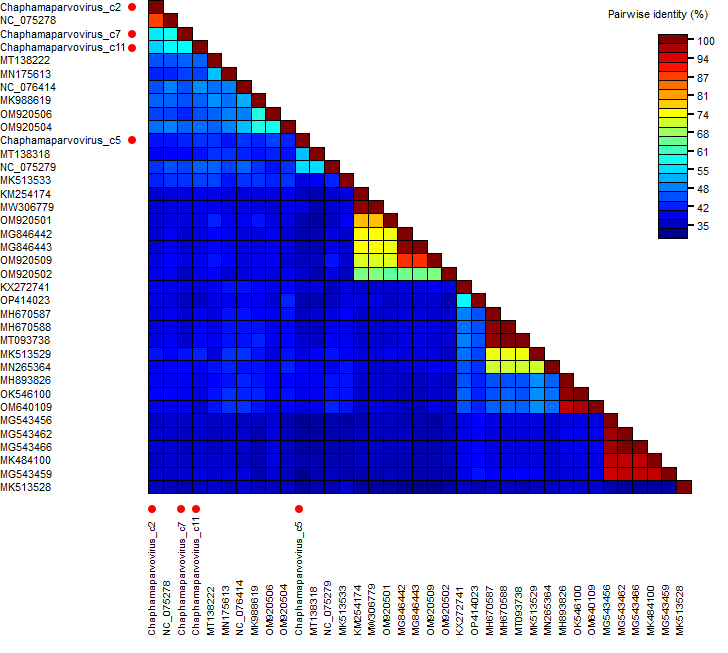

Supplement: Supplementary file 1 [file viruses-15-02068-s001.zip › Figure S1.jpg]

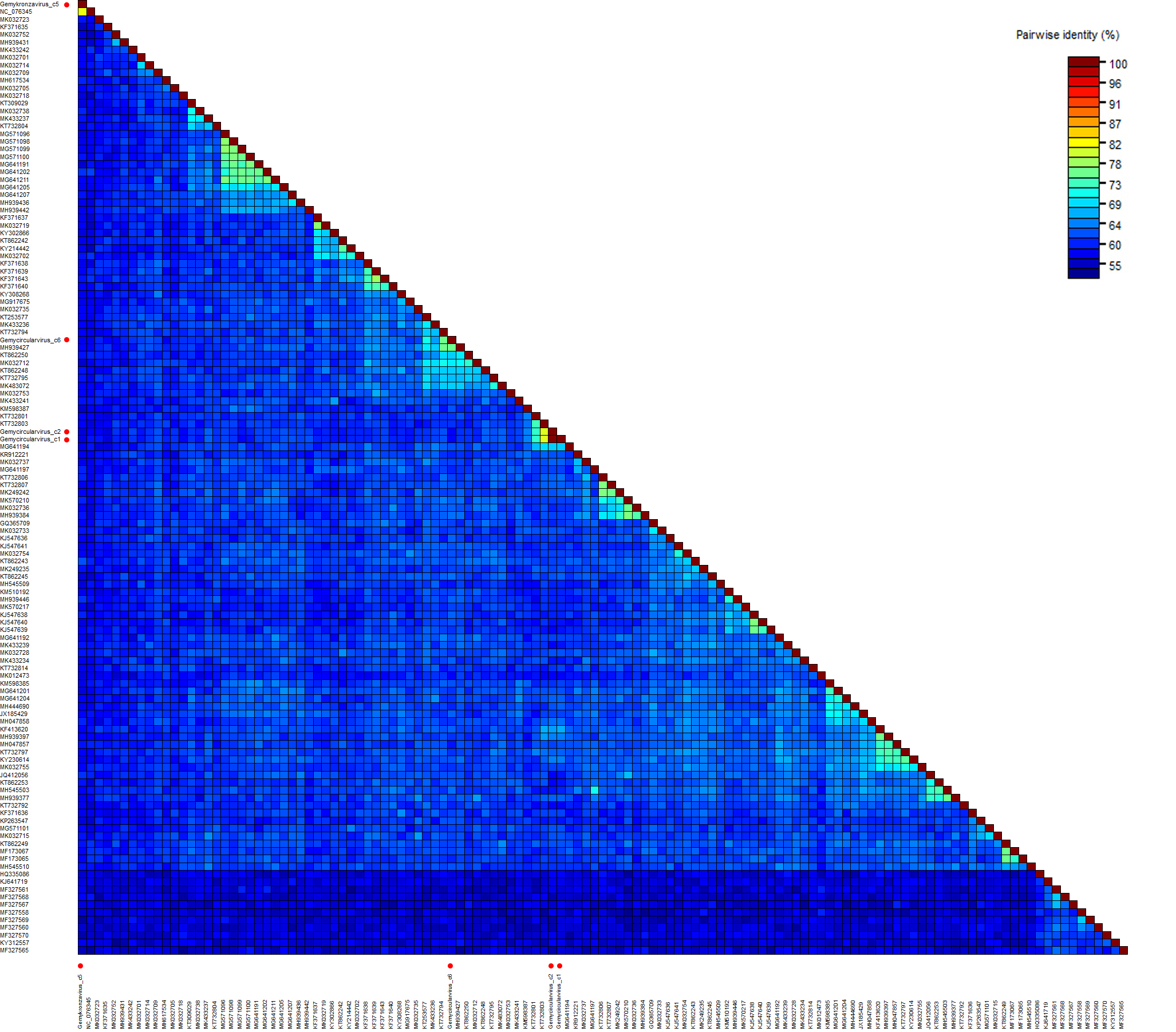

Supplement: Supplementary file 1 [file viruses-15-02068-s001.zip › Figure S2.jpg]
